# Efficacy of Pre- and Post-Treatment by Topical Formulations Containing Dissolved and Suspended *Silybum marianum* against UVB-Induced Oxidative Stress in Guinea Pig and on HaCaT Keratinocytes

**DOI:** 10.3390/molecules21101269

**Published:** 2016-09-22

**Authors:** Pálma Fehér, Zoltán Ujhelyi, Judit Váradi, Ferenc Fenyvesi, Eszter Róka, Béla Juhász, Balázs Varga, Mariann Bombicz, Dániel Priksz, Ildikó Bácskay, Miklós Vecsernyés

**Affiliations:** 1 Department of Pharmaceutical Technology, Faculty of Pharmacy, University of Debrecen, Nagyerdei körút 98, 4032 Debrecen, Hungary; feher.palma@pharm.unideb.hu (P.F.); ujhelyi.zoltan@pharm.unideb.hu (Z.U.); varadi.judit@pharm.unideb.hu (J.V.); fenyvesi.ferenc@pharm.unideb.hu (F.F.); roka.eszter@pharm.unideb.hu (E.R.); vecsernyes.miklos@pharm.unideb.hu (M.V.); 2 Department of Pharmacology and Pharmacotherapy, Faculty of Medicine, University of Debrecen, Nagyerdei körút 98, 4032 Debrecen, Hungary; juhasz.bela@pharm.unideb.hu (B.J.); varga.balazs@pharm.unideb.hu (B.V.); bombicz.mariann@pharm.unideb.hu (M.B.); priksz.daniel@pharm.unideb.hu (D.P.)

**Keywords:** silymarin, antioxidants, transcutol, sucrose esters, topical formulations, UVB-induced oxidative stress, HO-1 enzyme

## Abstract

Plants with high amounts of antioxidants may be a promising therapy for preventing and curing UV-induced oxidative skin damage. The objective of this study was to verify the efficacy of topical formulations containing dissolved and suspended *Silybum marianum* extract against UVB-induced oxidative stress in guinea pig and HaCaT keratinocytes. Herbal extract was dissolved in Transcutol HP (TC) and sucrose-esters were incorporated as penetration enhancers in creams. Biocompatibility of compositions was tested on HeLa cells and HaCaT keratinocytes as in vitro models. Transepidermal water loss (TEWL) tests were performed to prove the safety of formulations in vivo. Drug release of different compositions was assessed by Franz diffusion methods. Superoxide dismutase (SOD), catalase (CAT), glutathione peroxidase (GPx) and lipid peroxidation (MDA) activities were evaluated before and after UVB irradiation in a guinea pig model and HaCaT cells. Heme oxygenase-1 (HO-1) enzyme activity was measured in the epidermis of guinea pigs treated by different creams before and after UVB irradiation. Treatment with compositions containing silymarin powder (SM) dissolved in TC and sucrose stearate SP 50 or SP 70 resulted in increased activities of all reactive oxygen species (ROS) eliminating enzymes in the case of pre- and post-treatment as well. Reduction in the levels of lipid peroxidation end products was also detected after treatment with these two compositions. Post-treatment was more effective as the increase of the activity of antioxidants was higher. Lower HO-1 enzyme levels were measured in the case of pre- and post-treatment groups compared to control groups. Therefore, this study demonstrates the effectiveness of topical formulations containing silymarin in inhibiting UVB irradiation induced oxidative stress of the skin.

## 1. Introduction

*Silybum marianum* (Milk thistle) is a well-known medicinal plant that has been used for centuries as a herbal medicine for the treatment of liver-related diseases [1]. Many reviews have been published focusing on the determination and separation of active compounds in this herb [2]. Flavonolignan and non-flavonolignan components were separated from the *Silybum marianum* complex by a high performance liquid chromatography/tandem mass spectrometry (HPLC-MS/MS) method [3]. The major bioactive flavonolignans include silychristins A and B, silydianin, silybin A and B, isosilybin A and B. Furthermore, three additional components were also detected and partly separated, presumably two silybin stereoisomers and one isosilybin stereoisomer [4]. These components possess a wide range of antioxidant, cell regenerating and anticancer effects in the case of peroral application. [5]. Several articles have dealt with the topical application of *Silybum marianum* extract, mainly on the protective effects of silymarin and silybin against UVB-induced skin damage [6]. Overexposure of the skin to UV radiation causes sunburn, inflammation, oxidative stress, DNA damage, suppression of the immune system and skin cancers [7]. Both UVB (290–320 nm) and UVA (320–400 nm) induce the generation of ROS, which create oxidative stress in skin cells and play an essential role in the initiation, promotion and progression of skin aging and carcinogenesis [8].

Silybinin significantly decreased ROS production and apoptosis in HaCaT cells in a dose-dependent level after UVA irradiation [9]. To regulate ROS levels, the skin is rich in enzymatic (SOD, CAT, GPX) and non-enzymatic (reduced glutathione, ascorbic acid, ubiquinol and tocopherol) [10,11] antioxidant defense systems, thereby maintains physiological “own-redox” homeostasis.

UV-generated ROS affect the regulation of the gene expression of signaling molecules/cascades such as mitogen-activated protein-kinases and interrelated inflammatory cytokines as well [12]. Supporting the skin’s own protective mechanism by exogenous application of natural compounds (i.e., silymarin) is one approach for topical photoprotection [13]. Nucleotide excision repair mechanism is also responsible for the protective effect of silymarin on epidermal keratinocytes from ultraviolet radiation-induced apoptosis and DNA damage [14].

In additional to the classic antioxidant defense, the cytoprotective enzyme heme oxygenase exhibits antioxidant properties. HO-1 cleaves heme into three products: biliverdin, CO, and ferrous iron. Biliverdin is further reduced to an antioxidant effector molecule bilirubin [15,16]. Induction of HO-1 also a potent anti-inflammatory intracellular mediator and can cause anti-apoptotic effect as well [16].

Topical silymarin was demonstrated to have remarkable antitumor effects [17]. The molecular mechanism of inhibition of photocacinogenesis by cutaneous silymarin is based on its anti-oxidant and anti-inflammatory effects. Additionally, many studies have also reported on the effect of silymarin on UVB-induced suppression of immune responses and DNA damaging effects in the skin [18]. Cutaneous silymarin formulations may favorably supplement protection of sunscreens and may be useful in the treatment of skin diseases associated with UV radiation-induced inflammation and oxidative stress and skin cancer [19,20].

However, the low solubility and low bioavailability of silymarin limits extensive topical applicability [21]. Many methods and dosage forms have been described for optimizing these parameters [22,23]. As penetration enhancers surfactants are able to influence the barrier integrity of the skin and potentiate the transport mechanisms of active pharmaceutical ingredients (APIs) [24]. However, only a few articles have dealt with the potential of well-designed carrier systems, but the liberation and absorption of APIs may be affected by the use of ideal topical carriers [25]. Due to the relative impermeability of the stratum corneum, extensive preformulation studies are necessary in order to develop topical vehicles with optimal drug release and skin penetration [26].

Therefore, the aim of the present study was to design new hydrophilic topical formulations containing SM. In the first stage of our experiment, different creams are prepared. The novelty of our formulations is the concomitant application of different surfactants (three sucrose esters, polysorbate 60 and Cremophor) and a solvent (Transcutol). These components may increase the bioavailability, solubility and penetration of SM. Texture analysis and in vitro release studies were performed to qualify the biopharmaceutical properties of our ten compositions. Appropriate viscosity/consistency of compositions was measured by texture analysis investigation. We conducted in vitro dissolution studies to compare SM dissolution from our cream compositions. Diffusion coefficients and release rates were also determined. These parameters refer to the kinetic profile of SM permeation through the skin. Many studies have reported on the toxicity and the irritancy of these applied surfactants and cream bases [27]. Biocompatibility tests were performed to certify the harmlessness of these compositions. The carrier systems containing SM were tested on HeLa cells and on HaCaT keratinocytes. In vivo irritancy tests (TEWL) were also performed to characterize epidermal barrier function 24 h after application of creams. Transepidermal water loss measurement is one of the standard methods used to analyse the integrity of skin barrier treated with topically applied substances.

Four compositions were selected on the basis of these biopharmaceutical and biocompatibility tests. In the second stage of our experiment, we measured the in vitro antioxidant activities of selected formulations (Compositions III and IV and VIII and IX) on HaCaT cells and in vivo guinea pig animal model to demonstrate the efficacy of our topical formulations against UVB irradiation-caused significant oxidative stress via generation of reactive oxygen species (ROS) and these ROS’ rapid removal by non-enzymatic and enzymatic antioxidants. Therefore, different enzyme activities (glutathione peroxidase, GPX; superoxide dismutase, SOD; catalase, CAT) were determined. Lipid peroxidase tests were also performed and the level of malondialdehyde (MDA) was determined. Finally, heme-oxygenase (HO-1) enzyme activity was also measured in skin tissue of guinea pigs before and after UVB irradiation, because induction of HO-1 may protect against the cytotoxicity caused by oxidative stress.

## 2. Results

### 2.1. Texture Analyzer Studies

Resistance of the different silymarin creams compositions (Table 1) showed different values during the compression tests. 

**Figure 1:**
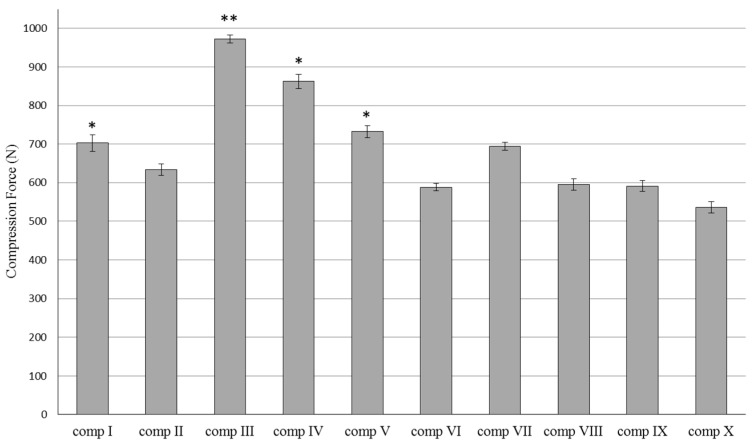
Resistance of silymarin cream formulations I–X, determined as compression force (N). Each data point represents the mean ± S.D., *n* = 5. Repeated-Measures ANOVA and for quantifying associations between the groups, Pearson correlation had been performed. Significant differences were marked in the figure with * (*p* < 0.01), and ** *(p* < 0.1), which show the significance levels in the case of compositions containing SM in suspended or dissolved forms.

The force (N) needed for the probe to penetrate into the creams is shown in Figure 1. Differences in compression force among the formulations I–X were measured. However, the resistance of creams containing SM in dissolved form with TC showed lower values compared to those formulations with the same emulgent where SM was in suspended form. Significant (*p* < 0.05) differences were detected between compositions I and V, III and VIII, IV and IX, V and X. Lower resistance values are more desirable for the sake of applicability. Higher compression force values were measured in the case of hard cream consistency. These hard composition structures may hinder the liberation of active pharmaceutical ingredients. Each data point represents the mean ± S.D., *n* = 5. Repeated-Measures ANOVA and for quantifying associations between the groups, Pearson correlation has been performed.

### 2.2. In Vitro Release Studies of Silymarin

**Figure 2:**
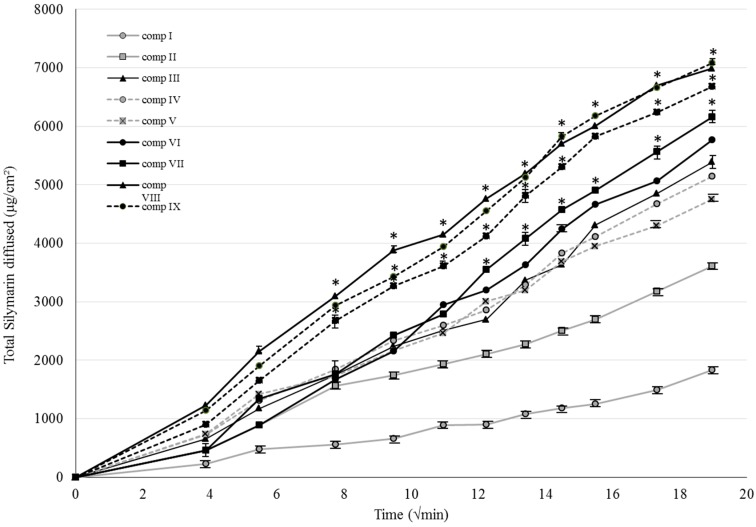
Release profiles of SM across isopropyl myristate (IPM) impregnated cellulose-acetate membrane of the formulations I–X. Each point represents the mean ± S.D. of five experiments. Repeated-Measures Anova and Pearson correlation tests were performed. Significant differences (*p* > 0.05) were marked with * in the figure. The asterisks show the significance levels in the case of compositions containing SM in suspended or dissolved forms.

Figure 2 shows the cumulative amount per unit area of silymarin diffused from the different formulations I–X against square root of time across isopropyl myristate (IPM) impregnated cellulose–acetate membrane. Each data point represents the mean ± S.D. of five experiments. Compositions in which SM was previously dissolved in TC resulted in the best diffusion values.

It was observed that the release of drug from the different formulae can be ranked in the following descending order: IX ˃ VIII ˃ X ˃ VII ˃ VI ˃ III ˃ IV ˃ V ˃ II ˃ I. Those creams that contained SM in dissolved form with the same emulgent showed higher diffusion values compared to suspended SM compositions. Significant differences (*p* < 0.05) could be detected between the dissolution of compositions II and VII, III and VIII, IV and IX, V and X. Our results showed that the drug release was better from compositions containing sucrose stearate SP50 or SP70 as emulsifying agent. The best diffusion was achieved from formulation IX, in which SM was dissolved in TC and the emulsifying agent was SP70, for which the diffused amount of API was 7083.47 ± 40.41 μg/cm^2^. Repeated-Measures ANOVA and for quantifying associations between the groups, Pearson correlation was been performed. Significant differences are marked in the figures with asterisks, which show the significance levels in the case of compositions containing SM in suspended or dissolved forms.

Silymarin release rate, k was determined from the slope of the amount of drug released per unit area versus the square root of time. The apparent diffusion coefficient (D) of the drug within the vehicle was estimated from the release rate value. Release rates and diffusion coefficient values are listed in Table 2 [28,29]. Significant differences (*p* < 0.05) could be seen between the diffusion coefficient values of compositions II and VII, III and VIII, IV and IX, V and X. Repeated-Measures ANOVA and Pearson correlation tests were performed. Significant differences are marked with asterisks in the figure, showing the significance levels in the case of compositions containing SM in suspended or dissolved forms.

### 2.3. MTT Cytotoxicity Tests

**Figure 3:**
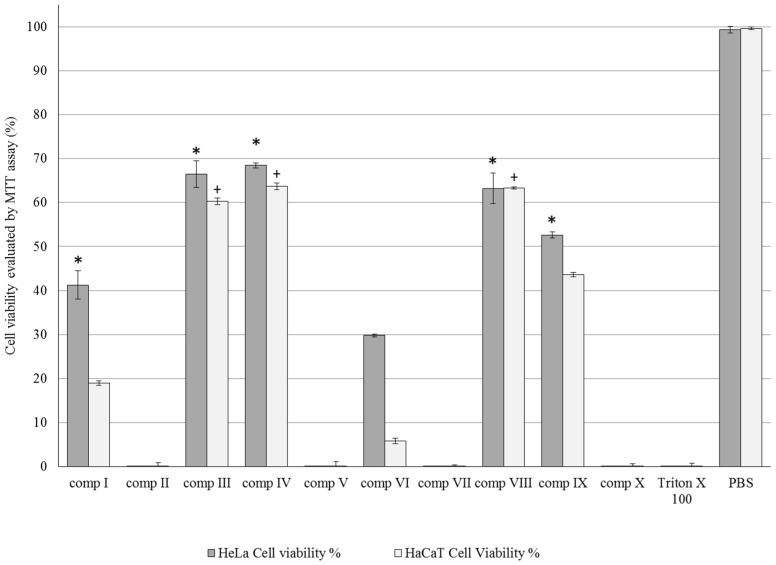
Cell viability evaluation following MTT assay on HaCaT and HeLa cells treated with compositions I–X Each data point represents the mean ± S.D., *n* = 10. Repeated-Measures ANOVA and Pearson correlation tests were performed. Significant differences (*p* > 0.05) are marked with * and crosses in our designed figures. * and + show the significance levels in the case of compositions I–X compared to the positive (Triton-X) and negative (PBS) controls.

Compositions I–X were tested with MTT cytotoxicity tests on both HeLa and HaCaT cell lines. Each composition was more toxic to HaCaT cells. Cell viability values ranked in the following descending order among the compositions containing emulgents SP 70 > SP 50 > P60 > PS 750 = CRC, for both the HeLa and HaCaT cell line. SP 70 was less toxic among the emulgents, the cell viability was 48.05% ± 0.6% on HeLa and 53.7% ± 0.6% on HaCaT cells. Those compositions which contain sucrose esters (SP 50 and 70, compositions III and IV, VIII and IX) as emulsifying agent resulted in satisfactory viability values. The cell viability values were compared to the positive control Triton × 100. A significant (*p* < 0.05) increase was detected in compositions I, III, IV, VIII and IX on HeLa cells and III, IV, VIII on HaCaT keratinocytes. Each data point represents the mean ± S.D., *n* = 10. Repeated-Measures ANOVA and Pearson correlation tests were performed (Figure 3).

### 2.4. In Vivo TEWL Measurements

**Figure 4:**
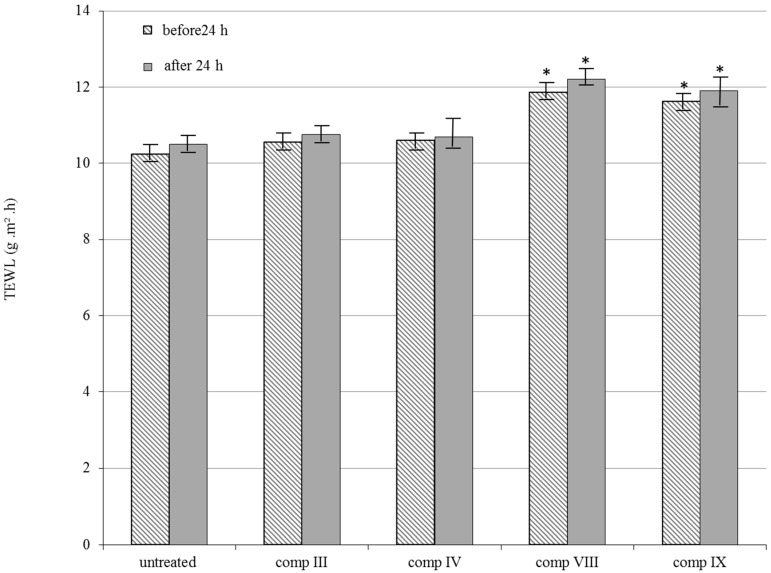
In vivo skin irritation examination. Changes in TEWL value before and after 24 h application of the selected silymarin creams III, IV, VIII, IX on the dorsal skin of guinea pigs. Each data point represents the mean ± S.D., *n* = 5. Repeated-Measures ANOVA and Pearson correlation tests were performed. Significant differences were marked with *, which show the significance levels in the case of compositions VIII and IX before and after 24 h compared to the untreated controls.

Among bioengineering methods TEWL measurement was chosen for evaluating the in vivo safety of formulations III, IV, VIII and IX on dorsal skin of guinea pig. Rates of TEWL increase in proportion to the level of damage in the skin so that high values correspond to greater toxicity. The difference in TEWL values before and after 24 h application of the formulations is shown in Figure 4. Preparations VIII and IX resulted in a greater TEWL values compared with the other formulations. However no significant increase was observed before and after 24 h application for either of the formulations.

### 2.5. In Vitro Antioxidant Activity on HaCaT Cell Line

**Figure 5:**
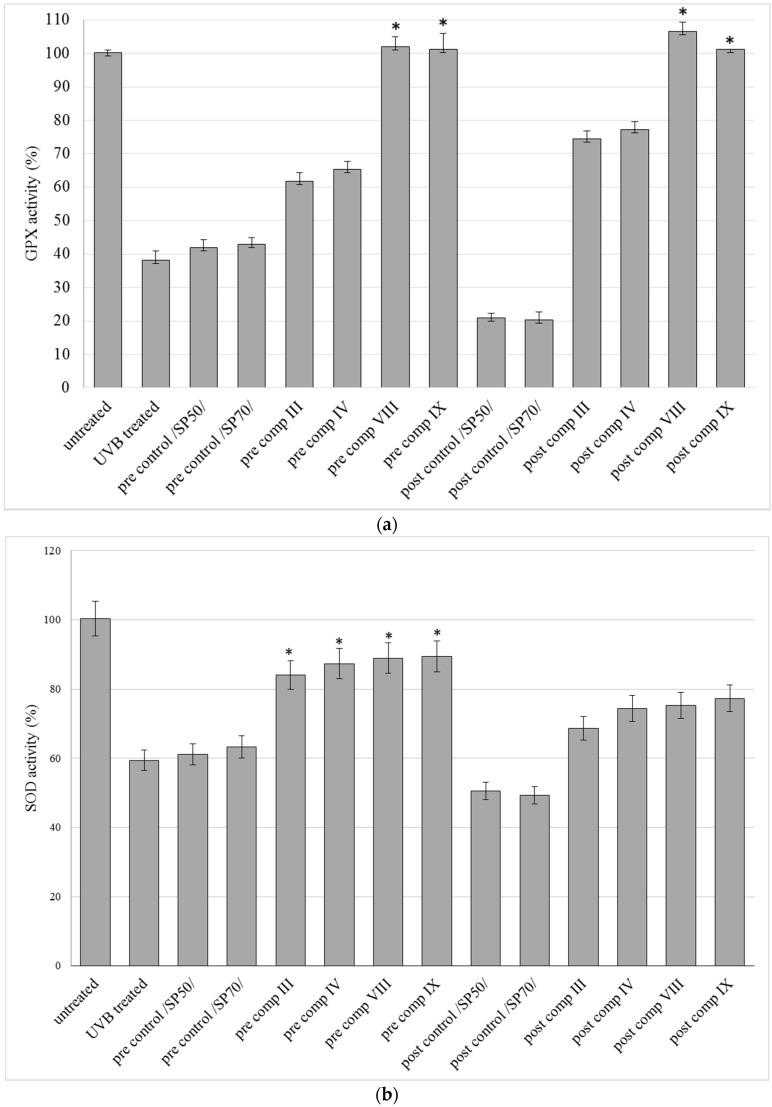

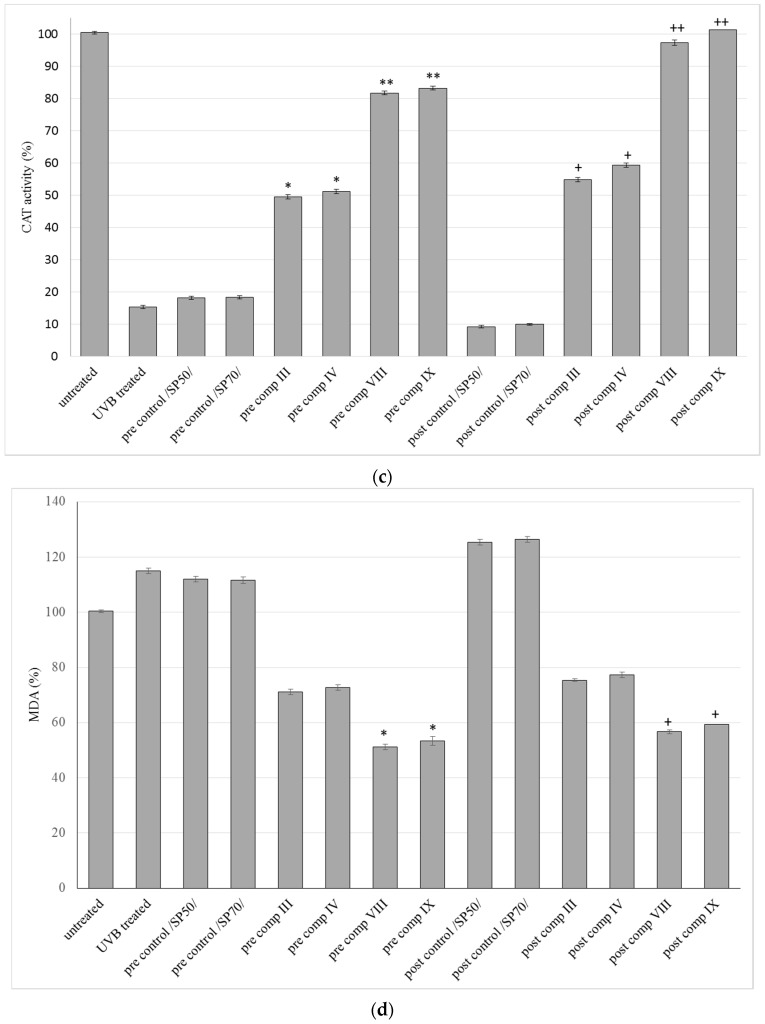
Effects of pre- and post-treatment with compositions III, IV, VIII, and IX on different antioxidant enzyme activities in HaCaT cell line receiving acute UVB irradiation. GPX (**a**); SOD (**b**); CAT (**c**) and MDA (**d**) activities are expressed as % of untreated keratinocytes. Control compositions without SM were: pre/post controls SP 50, SP 70. Data are expressed as mean ± S.D. *n* = 10. In the case of GPX, SOD, CAT, MDA evaluations Repeated-Measures ANOVA and Pearson correlation tests were performed. Significant differences were marked with * and + in the figures, showing the significance levels in the case of compositions containing SM in suspended or dissolved forms.

The effect of SM cream formulations III, IV, VIII, and IX against UVB irradiation was examined on the HaCaT cell line. Figure 5 shows the results of in vitro antioxidant activity measurements. The GPx (Figure 5a), SOD (Figure 5b) and CAT (Figure 5c) activities were decreased in control groups and in UVB treated groups as well after UVB irradiation compared to the untreated group. The pre-and also the post-treatment with the compositions increased the antioxidant activity of the enzymes.

Differences among the compositions could be observed as well: cream bases containing SM in dissolved or in suspended form prevented better the UV-B induced decrease in antioxidant activity of skin tissues in these three antioxidant tests compared to those controls which do not contain active substances (pre/post controls SP 50, SP 70). GPX level increased significantly (*p* < 0.05) in both pre- and post-treatment of the compositions compared to control groups. Significant (*p* < 0.05) increases were also detected after the pretreatment with compositions III, IV, VIII and IX in the level of SOD, but no significant (*p* < 0.05) increase was shown in CAT levels compared to control groups. The level of MDA (Figure 5d) in control groups and in the UVB-treated group increased after UVB exposure compared to the untreated group. It was observed that pre-treatment and post-treatment with compositions III, IV, and VIII, IX decreased the level of MDA, however compositions VIII, and IX, caused more reduction in the end product of lipid peroxidation. In the pretreatment and posttreatment of these preparation (VIII, IX) significant (*p* < 0.05) decreases were observed compared to control groups.

### 2.6. Antioxidant Activity on Guinea Pig Model

**Figure 6:**
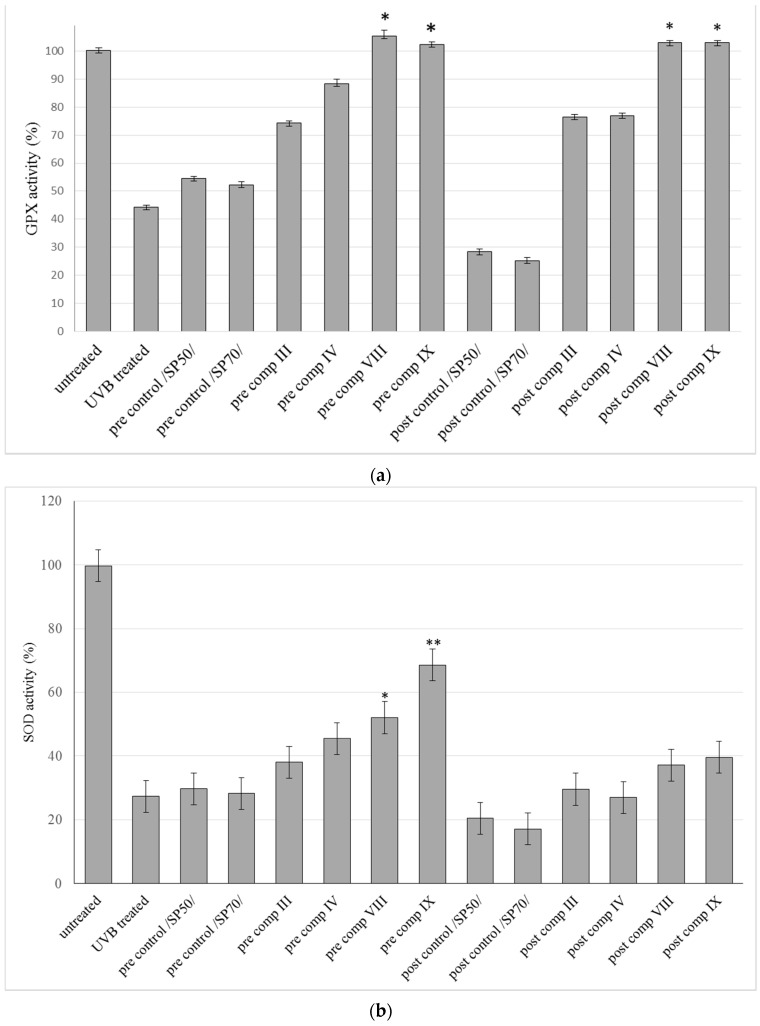

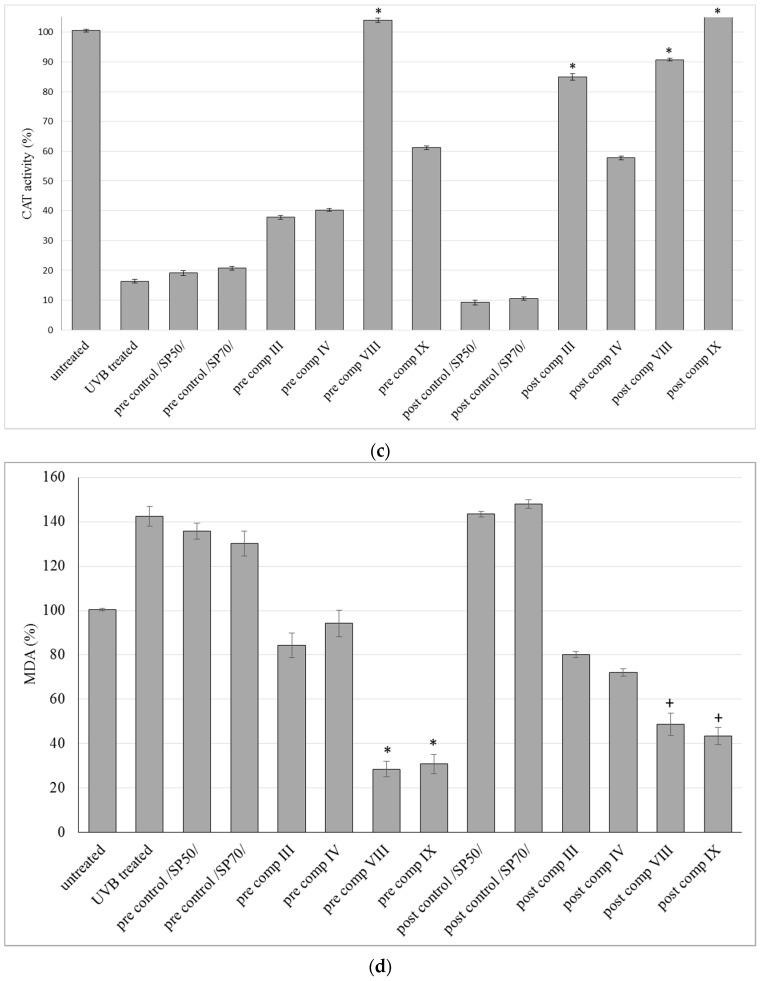
Effects of topical formulations containing silymarin in pre- and post-treatment on different antioxidant enzyme activities in dorsal skin tissue homogenate induced by UVB irradiation. GPX (**a**); SOD (**b**); CAT (**c**) and MDA (**d**) activities are expressed as % of enzyme activity of untreated guinea pigs. Bars represent means ± S.D., *n* = 5. In case of GPX, SOD, CAT, MDA evaluations Repeated-Measures ANOVA and Pearson correlation tests were performed. Significant differences are marked with * and + in the figures, showing the significance levels in the case of compositions containing SM in suspended or dissolved forms.

Cream formulations (III, IV, VIII, IX) containing SP 50 and SP 70 as emulsifying agents and SM in suspended or in dissolved form with the help of TC were selected for further in vivo antioxidant experiments according to the results of drug release studies, MTT cytotoxicity tests and in vivo TEWL measurements. The results of antioxidant enzyme activity measurement of GPx, SOD and CAT in epidermis of guinea pigs from different groups are summarized in Figure 6a–c. UVB exposure induced depletion of these antioxidant enzymes in UVB treated and control groups compared to untreated groups.

Activity assays of antioxidant enzymes from tissue samples showed that GPx, SOD and CAT activity was higher in the pre-treated and post- treated groups than in the control groups. In addition, formulations containing TC resulted in higher antioxidant enzyme activities compared to the formulations where SM was suspended in the creams. Significant (*p* < 0.05) increase was observed both in GPx and CAT activity in pre comp VIII, post comp VIII and post comp IX groups compared to control groups. Also significant (*p* < 0.05) increase could be detected in GPx activity in pre comp IX group compared to control group. Beside these results, CAT activity also showed significant (*p* < 0.05) increase in post comp III group. 

The SOD levels also increased significantly (p < 0.05) in two groups (pre-comp VIII and pre- comp IX) compared to control groups. Figure 6d shows variation in MDA concentration between groups during our experiments. Elevations in the level of end products of lipid peroxidation in the UVB exposed control and UVB treated groups compared to untreated group were registered. In our experiments SM cream formulations caused a significant reduction in MDA levels in the pre-treated and also in the post-treated groups compared to control groups. Lower significant (*p* < 0.05) MDA levels were detected in the comp VIII and composition IX groups both in pre- and post-treatment.

### 2.7. HO-1 Enzyme Activity

**Figure 7:**
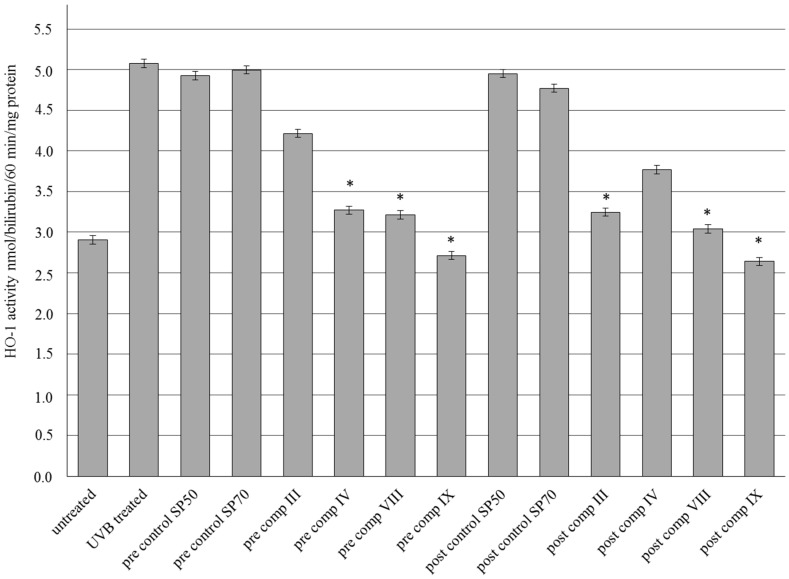
Effects of topical formulations containing silymarin in pre- and post-treatment on HO-1 enzyme activity in dorsal skin tissue homogenate of guinea pig induced by UV-B irradiation. HO-1 activity is expressed in nmol/bilirubin/60 min/mg protein. Bars represent means ± S.D., *n* = 5. In case of HO-1 evaluations Repeated-Measures ANOVA and Pearson correlation tests were performed. Significant differences compared to the untreated control are marked with * in the figures.

Four compositions of SM formulations (III, IV, VIII, IX) were selected for the experiments according to the results of drug release studies, MTT cytotoxicity tests and in vivo TEWL measurements. Figure 7 shows the results of HO-1 enzyme activity in epidermis of guinea pigs in pre- and post-treatment both before and after UVB irradiation. UV-B exposure resulted in a marked increase in HO-1 enzyme levels in UVB treated and control groups compared to untreated group. However the enzyme levels decreased both in pre- and posttreatment of the preparations. Significant (*p* < 0.05) decreases were observed in pre-comp IV, VIII and IX and post-comp III, VIII and IX groups. Asterisks show the significance levels compared to untreated group.

## 3. Discussion

In the present study, it was observed that topical compositions formulated with different penetration enhancers and containing *Silybum marianum* suspended or dissolved in Transcutol, inhibited cellular oxidative stress in the case of pre- and post-treatment in the hairless skin of guinea pig and in HaCaT cells irradiated with UVB. UVA and UVB irradiation to skin resulted in oxidative stress, and influenced the enzymatic and non-enzymatic ROS species levels [12,18]. ROS may play important roles in photocarcinogenesis, inflammation and immune suppression as tumor initiators, tumor promoters and inflammatory cytokine activators [30,31,32,33]. Topical supplementation of antioxidants can provide additional protection to neutralize these ROS species both from exogenous and endogenous sources [34].

Topical treatment with silymarin inhibits UV-induced oxidative stress in mouse models [35]. The main antioxidant components of silymarin are silibinin, silydianin and silychristin. The drawback of topical SM dosage formulation is the low water solubility of silymarin. The different lipophilicity of these active substances was investigated in the preformulation stage, but it just has a minor effect on skin permeation [21,36]. In our investigation, silymarin was dissolved in TC because TC is able to dissolve silymarin (350.1 mg/mL) and is a very strong penetration enhancer. Subsequently, TC may affect the skin penetration and bioavailability of SM compositions. TC is a good solubilizing agent as well as being non-toxic and biocompatible with the skin [37]. Generally, carrier systems can promote the penetration and absorption of APIs through different barrier systems (i.e., dermal barrier) [38]. Nevertheless, the design of these vehicles is a crucial point in drug formulation and medicine development [24]. To develop optimal carrier systems and promote SM penetration via the skin different non-ionic, amphiphilic tensides were selected for our ten compositions: P60, CRC, sucrose stearate SP 50, SP 70, and SP 750. These surfactants may play a role in the solubilisation and may also modify the bioavailability of drugs [24]. In vitro release studies provided information about SM dissolution from the carrier systems. Release rate differences were observed among the formulations I–X, and compositions with sucrose esters SP 50 and SP 70 (III, IV and VIII, IX) resulted in the highest diffused SM values. Outcomes of texture analysis studies confirmed that these creams showed optimal consistency values [39]. Although, these surfactants resulted in good diffusion values, determination of safety and non-toxic concentration is important for their application [27]. Biocompatibility investigations (MTT-tests) were performed on HeLa and HaCaT cells to verify the tolerability of cream compositions. HeLa cells represent a reliable model for testing topical preparations [40,41] while HaCaT cells are suitable to assess skin irritancy potential of excipients [42]. In the MTT cell viability test, sucrose-esters SP 50 and SP 70 proved to be more tolerable than Polysorbates and Cremophors in compositions. Compositions III, IV, and VIII, IX containing these tensides (SP 50 and 70) did not reduce HeLa and HaCaT cell viabilities under 50%. Since in vitro cytotoxicity data alone does not necessarily predict in vivo issues [43] irritancy tests were also performed. Results of animal skin irritancy test verified that our topical preparations containing silymarin, different surfactants and TC were highly tolerated, but after evaluation of the preformulation study data (in vitro dissolution study, texture analysis study, in vitro (MTT-cell viability) tests), four compositions (III, IV, VIII and IX) were selected for further in vivo antioxidant tests on HaCaT cells and in guinea pigs. Surfactants below or under their CMCs can raise the solubility of poorly dissolved APIs (i.e., SM), but may also influence the dissolution and the tolerability of active components [44]. In our study formulations containing sucrose esters SP 50 and SP 70 increased the drug solubility and drug permeability and showed good biocompatibility results.

Topical application of silymarin causes depletion of catalase and induction of cyclooxygenase in mouse model: application of these preparations to mouse skin prevents UV-induced oxidative stress [8]. In the present study, it was observed that every preparation containing sucrose stearate SP 50 or SP 70 increased CAT, SOD and GPx and decreased MDA activities compared to the controls. Post-treatment of suspended and dissolved silymarin produced the same effect as pre-treatment with different compositions. Dissolved silymarin in TC (VIII, IX) caused a more significant increase in the activity of ROS eliminating enzymes in the case of post-treatment. Reduction in the levels of end products of lipid peroxidation were registered after treatment with these four compositions (III, IV, VIII, IX). Treatment with compositions VIII and IX resulted in the greatest increase of ROS species, however the level of GPx was the highest in the case of pre- and post-treatment. It can be explained that penetration of dissolved SM in TC was better than with compositions III and IV containing suspended SM. Higher solubility of SM may influence the penetration and the efficacy of topical formulations [45]. GPx is a non-enzymatic antioxidant and maintain pro-oxidant/anti-oxidant balance resulting in cell and tissue stabilization [12]. Those formulations that contain sucrose esters SP 50 and SP 70 may promote all ROS species at a different rate but the promotion of non-enzymatic GPx may be more intensive. It was assumed that our in vitro data presented tight correlation with in vivo parameters as there were an increase in activity of CAT, SOD and GPx levels compared to the controls. Elevations in the levels of end products of lipid peroxidation were also measured on HaCaT cells. Svobodova et al. [8] confirmed that the flavonolignan components of silymarin suppressed oxidative stress caused by UVA and was useful in the treatment of UVA-induced skin damage. Silymarin showed a dose-dependent protective effect against UV-induced damage in human keratinocytes via inhibition of NF-κB activation [46]. Higher levels of SOD and CAT were measured on HaCaT in the case of pre- and post-treatment than in guinea pig. These enzymatic ROS level activations may be more expressed on keratinocytes due to the different effect of surfactants. The same tendency was observed on Caco-2 cell lines treated by blends of surfactants due to the modulation of tight junction proteins [24]. Different correlations were estimated between in vitro cell culture experiment data and in vivo animal experiment [43].

Marked decrease in the ROS species may refer UVA and UVB-induced oxidative stress. However, the work of Katiyar et al. [18] showed that topical application of SM inhibits UVB-induced inflammatory responses and photocarcinogenesis in mice as well. Nevertheless, high increase of HO-1 enzyme levels also represents stress against different environmental factors (i.e., UVB-induced photodamage, and cellular stressors [35,47]. Many mechanisms and factors of antioxidant and antiphotodamage activities of SM were widely studied [7]. However, there is no work which evaluates the effect of topical formulation of SM on the modification of HO-1 enzyme activity. Both UVA and UVB irradiation can lead to high levels of HO-1 expression of cells and are involved in an adaptive protective response against oxidative damage and it is considered a marker of cellular oxidative stress [35,47]. In our study, high increases of HO-1 enzyme activities were measured after UVB irradiation. Interestingly, topical administration of SM did not affect the HO-1 activity compared to the untreated control. It might be supposed that the high activity of antioxidant enzymes by the rapid elimination of free radicals may influence the activation of HO-1 enzyme systems in the skin. SM supplemented with penetration enhancers may be a potential in topical anti-inflammatory and antioxidant therapy to the skin based on the impact of ROS species activation and moderation of HO-1 enzyme activity. Identification and characterization of pharmacological compounds that induce HO-1 in a cell-specific and cell context-specific manner deserve further attention [48].

## 4. Materials and Methods

### 4.1. Materials

SM from *Silybum marianum* seeds was prepared according to Kahol et al. [49]. The SM did not contain any solvent residue. The same bioactive flavonolignans were determined as in the standards with the help of a HPLC-MS method [4]. P60 was obtained from Sigma-Aldrich Buchs (St. Gallen, Switzerland). TC was a kind gift from Gattefossé (Lyon, France). Cremophor A6, A25 was obtained from BASF (Ludwigshafen, Germany). Sucrose esters (SP 50, SP 70, PS 750) were kind gifts from Sisterna (Roosendaalc, The Netherlands). 3-(4,5-Dimethylthiazol-2-yl)-2,5-diphenyltetrazolium bromide (MTT), Dulbecco’s Modified Eagle’s Medium (DMEM), Hank’s Balanced Salt Solution (HBSS), phosphate buffered saline (PBS), Trypsin-EDTA, Heat-inactivated fetal bovine serum (FBS), l-glutamine, non-essential amino acids solution, penicillin-streptomycin, were purchased from Sigma-Aldrich). 96-Well cell plates, 12 well cell plates and culturing flasks were obtained from Corning (Corning, New York, NY, USA). Cetostearyl alcohol, stearic acid, propylene glycol, IPM, nipagin M were supplied by Hungaropharma Ltd., (Budapest, Hungary). HeLa cells (human cervical cancer cells) were obtained from the European Collection of Cell Cultures (ECACC, Public Health England, Salisbury, UK). HaCaT cells (human keratinocyte cells) were obtained from Cell Lines Service (CLS, Heidelberg, Germany).

### 4.2. Formulation of Topical Ointments

Different emulgents were used for the formulations: P60, CRC and sucrose esters (SP 50, SP 70 and PS 750). The oil-in water creams were produced by melting (60 °C) cetostearyl alcohol, stearic acid and IPM and mixed in order to prepare the oil phase of the preparation. The aqueous phase containing propylene-glycol, emulsifying agent, glycerol and purified water was heated up to the same temperature (60 °C) and mixed with the oil phase, homogenized and cooled down to 25 °C. Then, nipagin M was mixed in the preparation and finally, the active substance, SM. Final concentration of the powder incorporated in the creams was 5% either in suspended form (compositions I–V) or in dissolved form, previously dissolved with the help of TC (compositions VI–X) (Table 1).

### 4.3. Texture Analyzer Studies

The resistance of cream formulations was measured by a CT3 Texture Analyzer (Brookfield, Middleboro, MA, USA). Compression test as normal test was performed. The following parameters were fixed: trigger load (4 g), target (10 mm), speed (0.50 mm/s). A TA5 Cylinder type probe (12.7 mm diameter, and 35 mm length) was used during the test. All measurements were done in quintuplicate.

### 4.4. In Vitro Release Studies

Membrane diffusion and permeability studies were performed with a vertical Franz-diffusion cell system (Hanson Microette TM Topical and Transdermal Diffusion Cell System) [50]. Samples (0.3 g) were placed as donor phase on cellulose-acetate membrane (pore size 0.45 μm). Pre-treatment of the membrane by soaking in IPM was performed. Effective diffusion surface area was 1.767 cm^2^. 30% (*v*/*v*) alcohol was used as an acceptor phase in order to enhance the solubility of silymarin. Rotation of the magnetic stirrer was set to 450 rpm. The receptor medium was thermostated at 32 ± 0.5 °C throughout the experiment to imitate the temperature of physiological skin on the membrane in the Franz cell. Experiments were performed for 6 h. Samples of 0.8 mL were taken from the acceptor phase and replaced with fresh receiving medium [51]. Quantitative measurement of silymarin was carried out with an UV-spectrophotometer (Shimadzu, Tokyo, Japan) at a wavelength of λ = 287 nm. Calibration curve was determined before the spectroscopic measurements of silymarin. Linear connection was found between the concentration of silymarin and the measured absorbance. All experiments were performed in quintuplicates. 

### 4.5. Cell Cultures

HeLa [52] and also HaCat cell line was used in our experiments. HeLa cell is an immortalized cell line, derived from cervical cancer cells. HeLa cells were grown in plastic cell culture flasks in Dulbecco’s Modified Eagle’s Medium (Sigma-Aldrich Buchs), supplemented with 3.7 g/L NaHCO_3_, 10% (*v*/*v*) heat-inactivated fetal bovine serum (FBS), 1% (*v*/*v*) non-essential amino acids solution, 1% (*v*/*v*) l-glutamine, 100 IU/mL penicillin, and 100 IU/mL streptomycin at 37 °C in an atmosphere of 5% CO_2_.

The immortalized human keratinocyte line HaCaT [53] has close similarity in functional competence to normal keratinocytes. This cell line has been used in many studies as a paradigm for epidermal cells and therefore we selected HaCaT as the other cell model. HaCaT kertinocytes were grown in DMEM supplement with FBS (7%, *v*/*v*), streptomycin (100 U/mL), penicillin (0.1 mg/mL) and glutamine (4 mmol/L) in a humidified atmosphere with CO_2_ (5%; *v*/*v*) at 37 °C. Culture medium was changed twice a week for both cell types. 

Cells were subcultured following trypsinization. Cells were seeded at a density of 1 × 10^5^ cells/cm^2^ and grown near to confluence for experiments. The cells were routinely maintained by regular passaging. For cytotoxic and antioxidant experiments, cells were used between passage numbers 20 and 40.

### 4.6. MTT-Assay

The cytotoxic effects of compositions I–X was evaluated by a colorimetric cytotoxicity method i.e., the MTT test [54] HaCaT keratinocytes and HeLa cell lines were used for evaluation of the in vitro cytotoxic effects. The test was performed as follows: HaCaT and HeLa cells were seeded on flat bottom 96-well tissue culture plates at a density of 103 cells/well and allowed to grow in a CO_2_ incubator at 37 °C for 2–3 days. For these studies, the culture medium was removed, test solutions were added, and the cells were incubated for a further 30 min. After removing the samples, the cells were washed twice with 1 mL PBS, and another 3-h-incubation in a medium containing MTT at the concentration of 0.5 mg/mL followed. The dark blue formazan crystals were dissolved in acidic isopropanol (isopropanol:1.0 N hydrochloric acid = 25:1). Absorbance was measured at 570 nm against a 690 nm reference with a FLUOstar OPTIMA Microplate Reader (BMG LABTECH, Offenburg, Germany). Cell viability was expressed as the percentage of the untreated control [55]. Each experiment was repeated five times with five wells for each concentration.

### 4.7. In Vivo Skin Irritation Test

Before in vivo antioxidant tests, formulations III, IV, VIII, IX were tested for skin irritation. TEWL values were measured before and after 24 h application of the selected formulations to the dorsal skin of guinea-pigs. Cream (0.1 g) was spread uniformly over a sheet of non-woven polyethylene cloth (2 cm × 2 cm), which was then applied to the back area of a guinea-pig. Before the experiment the dorsal skin of animals were shaved properly. The polyethylene cloth was fixed with Tegaderm^®^ 3M adhesive dressing (3Med Kft, Budakeszi, Hungary), and Fixomull^®^ stretch adhesive tape (MEDIGOR Bt., Veszprém, Hungary). After 24 h, the cloth was removed, and the treated skin area was cleaned with a cotton wool swab. After withdrawal of the vehicle for 30 min, TEWL of the applied skin was measured with Tewameter^®^ (TM300, Courage & Khazaka, Köln, Germany). The temperature and relative humidity in the laboratory were kept at 26 °C and 55%, respectively. The number of samples for each experiment was five (*n* = 5).

### 4.8. UV-B Irradiation on HaCaT Cells, Pre-Treatment and Post-Treatment with Suspended and Dissolved Silymarin in TC

Antioxidant activity of compositions I–X was determined on HaCaT cells before and after UV-B exposure. The cells were seeded on flat bottom 12-well tissue culture plates at a density of 1 × 10^5^ cells/well and allowed to grow in a CO_2_ incubator at 37 °C for 2–3 days. In the pre-treatment group, culture medium was removed, test solutions i.e., SM in PBS and SM dissolved previously in TC, then diluted with PBS (SM + TC) were added, and the cells were incubated for further 20 min. After UV-B irradiation of 10 min. test solutions were removed, cells were washed with PBS and incubated for 24 h. In the post-treatment group, cells were first irradiated for 10 min, then treated with test solutions (SM and SM + TC) for 20 min. washed with PBS and incubated for 24 h. The following parameters were used for monitoring the antioxidant capacity of different compositions on UVB treated keratinocytes: SOD, CAT, GPx and malondialdehyde (MDA) activities. All experiments were performed in quintuplicate.

### 4.9. Experimental Animals

Male Hartley guinea-pigs weighing 250–350 g were used for evaluation of dermal antioxidant effects. The animals were fed regular rodent chow ad libitum with free access of water. The guinea-pigs were allowed 1 week to acclimatize before the experiments and were kept at 25 ± 2 °C, with a relative humidity of 55% ± 5% and a 12 h light-dark cycle. All animals included in the present study were handled and received humane care in compliance with the National Society for Medical Research and Guide for the Care and Use of Laboratory Animals prepared by the National Institutes of Health (No. 10/2014/DEMÁB, extended until 2019).

### 4.10. UV-B Irradiation, Pre-Treatment and Post-Treatment with Silymarin Cream Formulations, in Vivo

Pre-treatment and post-treatment with SM in different compositions of creams III, IV, VIII, IX were tested for antioxidant capacity before and after UV-B radiation. Animals were exposed to UV-B irradiation (acute exposure, one irradiation of 350 μW) from a distance of 20 cm. At the beginning of the experiment dorsal skin of guinea-pigs was shaved and covered with aluminum foil into which ten rectangular slots (0.8 cm × 1.3 cm) had been cut, next 0.1 g cream was applied to the skin under each slot [56]. For the antioxidant studies animals were divided into two groups of 10 animals each. Guinea-pigs in group I were pre-treated topically with the cream formulations and the vehicles (pre control SP 50 and pre control SP 70) for 20 min., then the animals were placed under the UV-B apparatus and the uncovered skin was irradiated for 10 min. Creams were washed 24 h after irradiation and skin samples were collected. In the II group, animals were first irradiated for 10 min., then post-treated topically with the cream formulations or vehicles (post control SP 50 and post control SP 70) and after 24 h creams were washed and skin samples were collected. At the end of experiment, animals were deep anesthetized with ketamine/xylazine (30–44 mg/kg, 1–5 mg/kg) and the tissue samples were dissected. Samples were perfused and rinsed with a PBS (phosphate buffered saline pH 7.4) solution to remove any red blood cells and clots. Skin samples were frozen with liquid nitrogen and kept at −80 °C until further processing. Tissues samples (0.8 cm × 1.3 cm) were cut from all the animals, immediately after the experiments, divided into four equal pieces for the determination of tissue SOD, CAT, GPx and MDA activities.

### 4.11. Antioxidant Activity

#### 4.11.1. Superoxide Dismutase Activity

Tissues were homogenized (Homogeniser, ×520, Ingenieurbüro CAT, M-Zipperer GmbH, Ballrechten-Dottingen, Germany) in 20 mM HEPES buffer (1 mM EGTA, 210 mM mannitol, and 70 mM sucrose/g tissue), pH 7.2. Then homogenate was centrifuged at 1500× *g* for 5 min at 4 °C, and the supernatant assayed for SOD activity using the Cayman assay kit (Cayman Chemical, Ann Arbor, MI, USA; www.caymanchem.com/pdfs/706002.pdf).

Cells (1 × 10^6^) were collected using a rubber policeman, centrifuged at 1000× *g* for 10 min at 4 °C. Cell pellet was homogenized in cold 20 mM HEPES buffer (1 mM EGTA, 210 mM mannitol, and 70 mM sucrose/g tissue), pH 7.2 centrifuged at 10,000× *g* for 15 min at 4 °C, and the supernatant assayed for SOD activity using the Cayman assay kit.

#### 4.11.2. Catalase Activity

Tissue samples were homogenized (Homogeniser, ×520) in cold buffer (50 mM potassium phosphate, pH 7.0, containing 1 mM EDTA) per gram tissue, centrifuged at 10,000× *g* for 15 min at 4 °C, and the supernatant assayed for CAT activity using the Cayman assay kit.

Cells (1 × 10^6^) were collected using a rubber policeman, centrifuged at 1000× *g* for 10 min at 4 °C. Cell pellet was homogenized on ice in 2 mL cold potassium phosphate buffer, pH 7.0, containing 1 mM EDTA per gram tissue, centrifuged at 10,000× *g* for 15 min at 4 °C, and the supernatant assayed for CAT activity using the Cayman assay kit.

#### 4.11.3. Glutathione Peroxidase Activity

Tissue samples were homogenized (Homogeniser, ×520) in 10 mL cold buffer containing 50 mM Tris-HCL, pH 7.5, 5 mM EDTA and 1mM dithiothreitol, per gram tissue. The homogenate was then centrifuged at 10,000× *g* for 15 min at 4 °C. The supernatant was assayed for GPx activity using the Cayman assay kit.

Cells (1 × 10^6^) were collected using a rubber policeman, centrifuged at 1000× *g* for 10 min at 4 °C. Cell pellet was homogenized in cold buffer containing 50 mM Tris-HCL, pH 7.5, 5 mM EDTA and 1 mM dithiothreitol. The homogenate was then centrifuged at 10,000× *g* for 15 min at 4 °C. The supernatant was assayed for GPx activity using the Cayman assay kit.

#### 4.11.4. Lipid Peroxidation (MDA) Test

Tissue samples (10 mg) and cells (1 × 10^6^) were homogenized on ice in 300 µL of the MDA Lysis Buffer containing 3 µL of BHT (100×) centrifuged at 13,000× *g* for 10 min to remove insoluble material. The supernatant was assayed for Lipid Peroxidation (MDA) assay kit (Sigma-Aldrich, St. Louis, MO, USA). Lipid peroxidation was determined by the reaction of MDA with thiobarbituric acid (TBA) to form a colorimetric (532 nm) product, proportional to the MDA present. All antioxidant tests were performed in quintuplicate.

### 4.12. Measurement of HO-1 Enzyme Activity

Activity of heme oxygenase-1 enzyme in guinea pig skin tissue was measured. Tissue samples were homogenised in 200 mM phosphate buffer. pH 7.4. The supernatant was collected by centrifugation of the homogenate for 30 min at 20.000× *g* at 4 °C. Assessment of heme oxygenase activity was measured on each sample of supernatant according to protocol used by Tenhunen et al. [57]. Briefly, enzyme activity was performed with a computer-based spectrophotometric analysis of heme formation to bilirubin. HO-1 enzymatic assay used a reaction mixture containing, aliquot of the supernatant, plus glucose-6-phosphate 2 mM, glucose-6-phosphate dehydrogenase 0.14 U/mL, heme 15 μM, NADPH 105 μM, rat liver cytosol as a source of recombinant biliverdin reductase 120 μg/mL, MgCl_2_ 2 mM and KH_2_PO_4_ 100 mM. The reaction was made up to a final volume (2 mL) for each sample and was incubated at 37 °C for 1h in the dark. The reaction was arrested by placing the samples on ice. Chloroform was added to terminate the reaction and bilirubin was extracted following centrifugation, and measured by spectrophotometric method, reading the difference in absorbance between 460 and 530 nm. The HO-1 activities were expressed in nmol of bilirubin formed per 1 milligram of protein per hour [57].

### 4.13. Statistical Analysis

Data were analyzed using SigmaStat (version 3.1; SPSS, Chicago, IL, USA) and presented as means ± S.D. Comparison of the groups in texture analyzer studies, in vitro release studies, MTT cell viability assays., in case of SOD, CAT, MDA, GPx and HO-1 enzyme activity evaluations Repeated-Measures ANOVA and Pearson correlation tests were performed. Significant differences were indicated in the figures with asterisks and/or crosses. Differences were regarded as significant, with *p* < 0.05. All experiments were carried out in quintuplicate and repeated at least five times (*n* = 5).

## 5. Conclusions

In our present study, silymarin topical formulations containing different surfactants and penetration enhancers with high bioavailability were designed. These preparations showed higher antioxidant activity against UVB-induced oxidative stress in guinea pig and HaCaT cells as pre- and post-treatment compared to controls. Topical application of SM can influence sufficiently the level of HO-1 enzyme which is a factor of cellular stress. The efficacy of our cream formulations is based on the impact of ROS species activation and moderation of HO-1 enzyme activity. It can be concluded that silymarin cream may be effective natural topical formulation to prevent and treat the skin against UVB-irradiation

## Figures and Tables

**Table 1:** The composition of the silymarin creams.

Composition	Emulgents							Cetostearyl Alcohol (4.6 g) Stearic Acid (10 g) Propylene Glycol (5 g) IPM (5 g) Nipagin M (1 g) Purified water (ad 100 g)
	SM (5 g)	TC (14.2 g)	P60 (3 g)	CRC (3 g)	SP 50 (3 g)	SP 70 (3 g)	PS 750 (3 g)	
I	+		+					+
II	+			+				+
III	+				+			+
IV	+					+		+
V	+						+	+
VI	+	+	+					+
VII	+	+		+				+
VIII	+	+			+			+
IX	+	+				+		+
X	+	+					+	+

Abbreviations: SM (silymarin powder), TC (Transcutol HP), P60 (Polysorbate 60), CRC (Cremophor A6 and A25 in the ratio 1:1), SP 50 (sucrose stearate SP 50), SP 70 (sucrose stearate SP 70), PS 750 (sucrose stearate PS 750), IPM (isopropyl myristate).

**Table 2:** Silymarin release rate and the diffusion coefficient values of the compositions (I–X). Each data point represents the mean ± S.D., *n* = 5, *p* > 0.05. Repeated-Measures Anova and Pearson correlation tests were performed. Significant differences are marked with * in the table. * show the significance levels in the case of compositions containing SM in suspended or dissolved forms.

Composition	Release Rate	Diffusion Coefficient
	k·10^2^ (µg/cm^2^·min½) ± S.D.	D 10^5^ (cm^2^/min) ± S.D.
I	0.075 ± 0.12	0.21 ± 0.02
II	1.30 ± 0.32	0.73 ± 0.05
III	1.41 ± 0.33	1.36 ± 0.23
IV	2.01 ± 0.23	1.57 ± 0.25
V	4.24 ± 0.14	0.71 ± 0.23
VI	2.00 ± 0.31	0.93 ± 0.25
VII	5.94 ± 0.45	24.1 ± 0.24 *
VIII	4.68 ± 0.26	47.23 ± 0.42 *
IX	4.71 ± 0.13	30.22 ± 0.20 *
X	3.99 ± 0.35	18.00 ± 0.13 *
